# Type 1 and Type 2 Diabetes and Cancer Mortality in the 2002-2009 Cohort of 39 811 French Dialyzed Patients

**DOI:** 10.1371/journal.pone.0125089

**Published:** 2015-05-12

**Authors:** Adélaïde Pladys, Cécile Couchoud, Aurélie LeGuillou, Muriel Siebert, Cécile Vigneau, Sahar Bayat

**Affiliations:** 1 Département d’Epidémiologie et de Biostatistiques EHESP, Rennes, France; 2 Registre REIN, Agence de la biomédecine, La Plaine Saint Denis, France; 3 CHU Pontchaillou, service de néphrologie, Rennes, France; 4 Université Rennes 1, UMR CNRS 6290, Rennes, France; 5 EA MOS EHESP, Rennes, France; Department of Transplantation and Renal Medicine, AUSTRALIA

## Abstract

End-stage renal disease is a chronic and progressive pathology associated with several comorbidities, particularly diabetes. Indeed, diabetes is the first cause of end-stage renal disease and, in France, 42% of incident patients had diabetes in 2012. In the general population, diabetes is associated with increased cancer risk. The aim of this study was to examine the association between risk of cancer death and diabetes in a large French cohort of patients with end-stage renal disease. Data on all patients with end-stage renal disease who initiated dialysis in France between 2002 and 2009 were extracted from the Renal Epidemiology Information Network registry. The risk of dying by cancer was studied using the Fine and Gray model to take into account the competing risk of death by other causes. We analyzed 39 811 patients with end-stage renal disease. Their mean age was 67.7±15 years, 39.4% had diabetes and 55.3% at least one cardiovascular disease. Compared with the non-diabetic group, patients with diabetes were older and had more cardiovascular and respiratory comorbidities when they started dialysis. Conversely, fewer diabetic patients had also a tumor at the beginning of the renal replacement therapy. Cancer was indicated as the cause of death for 6.7% of diabetic and 13.4% of non-diabetic patients. The Fine and Gray multivariate analyses indicated that diabetes (HR=0.72 95% CI: [0.68-0.95], p<0.001) and also female gender, peritoneal dialysis, cardio-vascular disease and kidney transplantation were associated with decreased risk of death by cancer. In this French cohort of patients with end-stage renal disease, diabetes was not associated with a significant increased risk of dying from cancer. Studies on the incidence of cancer in patients with ESRD are now needed to evaluate the potential association between diabetes and specific malignancies in this population.

## Introduction

End-Stage Renal Disease (ESRD) is a chronic progressive disease and a major public health issue. During the last decades, the number of people treated for ESRD has dramatically increased worldwide and also in France [1, 2]. Renal replacement therapy (RRT) by hemodialysis, peritoneal dialysis or kidney transplantation is necessary to improve ESRD patients’ survival. In France, patients on dialysis, patients receiving kidney transplantation and the annual incident cases of RRT are recorded in the Renal Epidemiology Information Network (REIN) registry.

Chronic kidney diseases are associated with several comorbidities, such as cardiovascular complications and diabetes [1, 3–6], and with premature death [4; 6]. In France, among the patients who started RRT in 2012, 42% also had diabetes and diabetic nephropathy was the primary renal disease in 22% of these diabetic patients [7]. Moreover, in patients receiving RRT, cancer incidence is higher than in the general population [3–5, 8–16]. This could be due to several reasons, such as chronic infection, weakened immune system or DNA mutations [8, 12–16]. In France, 12% of patients with ESRD who started RRT in 2012 also had a tumor (including hematological malignancies). At 12/31/2012, 39% of incident patients who began a dialysis between 2002 and 2012 died and in 10% of these patients, death was related to cancer [17]. Type 2 diabetes is a common metabolic disease associated with increased cancer risk in the general population [5, 18–20]. Pancreas secretion disorders induce higher insulin levels that stimulate the Insulin Growth Factor (IGF) pathway [19–21]. Insulin and IGF are both mitogen agents that have carcinogenic effects and might be involved in metastasis development [19–21]. Besides insulin, new diabetes treatments could also influence cancer risk, although this notion is controversial [22].

On the other hand, few studies reported that among patients with chronic kidney disease [5] or ESRD [10–13], cancer rate is lower in individuals suffering also from diabetes than in patients without diabetes. However, some of these works focused specifically on a single cancer type, such as kidney [10, 11] or urinary tract tumors [11], or on a specific group of patients with ESRD [10], whereas the others did not take into account the patients’ comorbidities [12, 13].

As no study on the association of cancer and diabetes in dialyzed patients with ESRD has been conducted in France to date, we decided to evaluate the association between cancer mortality and diabetes in these patients. We chose to analyze cancer in terms of mortality because in the French REIN registry, the incidence of cancer is not a mandatory field. Conversely, the cause of death is an item that must be filled in by the nephrologist who followed the patient and can thus be considered highly reliable. In addition, the clinical research associates make sure that this item is properly specified in the case of death and, consequently, missing data for this category are not recorded. This allowed an exhaustive analysis of the cancer mortality rate in patients with ESRD in France.

## Materials and Methods

### Participants

This was a retrospective study, using data that had been prospectively collected in the REIN registry. We included all patients aged 18 years and over who started dialysis in the French regions contributing to the REIN registry between January 1, 2002 and December 31, 2009. The REIN registry has been progressively covering the entire French territory between 2002 and 2011 and 100% uptake of patients was reached in 2011. On December 31, 2009, the REIN registry covered 88% of the French territory (23 of the 26 French regions) [23]. Biological and clinical data are recorded in the REIN registry when a patient starts RRT (dialysis or preemptive kidney transplantation). Patients with unknown diabetic status, preemptive kidney transplantation or HIV-positive status were excluded (n = 3 785).

### Variable and data collection

Four categories of variables were studied: i) demographic data: sex and age; ii) biological and clinical data at dialysis initiation: comorbidities (type 1 and type 2 diabetes, cardiovascular diseases, tumors, cirrhosis, respiratory chronic diseases), smoking status (current smoker, former smoker, and never smoker), Body Mass Index (BMI) categorized as <23, 23–25 and >25kg/m^2^, hemoglobin (<10, 10–12 and >12g/dl) and serum albumin (<30 and ≥30g/dl); iii) treatment information: first dialysis modality (hemodialysis, peritoneal dialysis), date of the first dialysis and kidney transplantation during the RTT follow-up; iv) date and causes of death in six categories (heart diseases, renal diseases, cancer, diabetes, infectious diseases and other causes). When missing data were present, they were grouped in an independent category and included in the statistical analysis.

### Statistical analyses

Baseline characteristics were first described. Then, the characteristics of diabetic and non-diabetic patients were compared using the Chi-square test. The biological and clinical characteristics of these two groups were also compared after adjustment for age and sex.

The end of study was December 31, 2010 to allow an adequate follow-up of all patients and death was considered the clinical endpoint. Patient survival was assessed from the date of the first dialysis until death. The Fine and Gray survival model was used for these analyses to take into account the competing risk of death by other causes than cancer. All patients’ characteristics with a *P-value*< 0.2 in the univariate analysis were included in the multivariate Fine and Gray analysis. Results were given as hazard ratios (HR) and 95% confidence intervals (CI). A *P-value* <0.05 was considered statistically significant. For subgroup analyses the whole ESRD population with diabetes was divided in patients with type 1 diabetes or type 2 diabetes. Statistical analyses were performed with the Stata 11.1 (College Station, TX) software.

### Ethics statement

This retrospective study approved by the French Biomedecine Agency included patients’ informations which have been anonymized and de-identified directly in the database and before the extraction for analysis.

Subjects involved were extracted from the French REIN registry which received agreement from the CNIL (Commission Nationale de l’Information et des Libertés) in 2010. The REIN is registered with the CNIL with 903188 Version 3 number.

## Results

### Patients’ characteristics

Between 2002 and 2009, 39 811 patients aged over 18 years started dialysis in the 23 French regions contributing to the REIN registry (Table 1). The patients’ mean age at the beginning of RRT was 67.7 ± 15 years. More than 60% of them were males and hemodialysis was the first RRT for 87.7% of patients. At RRT initiation, more than half of all patients (55.3%) had at least one cardiovascular disease, 39.4% had diabetes, 2.2% cirrhosis, 11.2% a respiratory disease and 9.9% a cancer. At the end of the study (12/31/2010), 19 165 (48.14%) patients were dead. The main causes of death were cardiovascular complications (26.8% of patients), infectious diseases (12% of patients) and cancer (10.4% of patients).

**Table 1:** Characteristics of patients with ESRD at dialysis initiation.

Baseline characteristic	Total (n = 39 811)		Diabetics (n = 15 702)	Non-diabetics (n = 24 109)	
	n	%	n (%)	n (%)	p
**Sex**					<0.001
*Male*	24785	62.3	9442 (60.1)	15343 (63.6)	
*Female*	15026	37.7	6260 (39.9)	8766 (36.4)	
**Age (years)**					<0.001
*<60*	10772	27.1	3036 (19.3)	7736 (32.1)	
*60–75*	13711	34.4	6680 (42.6)	7031 (29.2)	
≥*75*	15328	38.5	5986 (38.1)	9342 (38.8)	
**Diabetes**					
*Yes*	15702	39.4	-	-	
*No*	24109	60.6	-	-	
**First treatment**					<0.001*
*Hemodialysis*	34901	87.7	13962 (88.9)	20939 (86.8)	
*Peritoneal dialysis*	4910	12.3	1740 (11.1)	3170 (13.2)	
**Smoking status**					<0.001*
*Current smoker*	3591	9.0	1109 (7.0)	2482 (10.3)	
*Never smoker*	19539	49.1	7549 (48.1)	11990 (49.7)	
*Former smoker*	9221	23.2	3859 (24.6)	5362 (22.2)	
*Missing*	7460	18.7	3185 (20.3)	4275 (17.8)	
**Cardio-vascular diseases** ^†^					<0.001*
*Yes*	22030	55.3	10671 (68.0)	11359 (47.1)	
*No*	17257	43.4	4652 (29.6)	12605 (52.3)	
*Missing*	524	1.3	379 (2.4)	145 (0.6)	
**Cirrhosis**					<0.001*
*Yes*	859	2.2	397 (2.5)	462 (1.9)	
*No*	38109	95.7	14736 (93.9)	23373 (97.0)	
*Missing*	843	2.1	569 (3.6)	274 (1.1)	
**Respiratory Disease**					<0.001*
*Yes*	4439	11.2	1993 (12.7)	2446 (10.1)	
*No*	34407	86.4	13069 (83.2)	21338 (88.5)	
*Missing*	965	2.4	640 (4.1)	325 (1.4)	
**Active malignancies** ^‡^					<0.001*
*Yes*	3947	9.9	1052 (6.7)	2895 (12)	
*No*	35010	88	14072 (89.6)	20938 (86.9)	
*Missing*	854	2.1	578 (3.7)	276 (1.1)	
**BMI (kg/m²)** ^s^					<0.001*
*<23*	9793	24.6	2353 (15.0)	7440 (30.9)	
*23–25*	5171	13.0	1660 (10.6)	3511 (14.5)	
*>25*	14266	35.8	7276 (46.3)	6990 (29.0)	
*Missing*	10581	26.6	4413 (28.1)	6168 (25.6)	
**Hemoglobin (g/dl)**					<0.001*
*<10*	12187	30.6	4826 (30.7)	7361 (30.5)	
*10–12*	13446	33.8	5202 (33.2)	8244 (34.2)	
*>12*	4277	10.7	1588 (10.1)	2689 (11.2)	
*Missing*	9901	24.9	4086 (26.0)	5815 (24.1)	
**Serum albumin (g/dl)**					<0.001*
*<30*	5318	13.4	2360 (15.0)	2958 (12.3)	
*≥30*	15651	39.3	5804 (37.0)	9847 (40.8)	
*Missing*	18842	47.3	7538 (48.0)	11304 (46.9)	

*After adjusting for sex and age

^†^Cardio-vascular diseases included: myocardial infarction, arrhythmias, coronary insufficiency, heart failure, lower limbs arteritis, cerebrovascular accident.

^‡^Solid tumors and hematological malignancies

^s^BMI: Body Mass Index

Among the patients with diabetes, 1356 (9.1%) had type 1 diabetes and 13 607 (90.9%) type 2 diabetes. Patients with diabetes were older (69.75 ± 11.4 years) than non-diabetic patients (66.42 ± 16.7 years, p<0.001) (Table 1) and the proportion of overweight (BMI>25kg/m^2^) individuals was higher in the diabetic group (46.3% vs 29% of non-diabetic patients, p<0.001). Most comorbidities were significantly more frequent in diabetic patients. Indeed, 68% of patients with diabetes also had cardiovascular diseases (vs 47.1% of non-diabetic patients, p<0.001), 2.5% had cirrhosis (vs 1.9% of non-diabetics patients, p<0.001) and 12.7% a respiratory disease (vs 10.1% of non-diabetic patients, p<0.001). However, only 6.7% of diabetic patients had a tumor at RRT initiation (vs 12% of non-diabetic patients, p<0.001). Comparison of the clinical characteristics in diabetic and non-diabetic patients was done after adjustment for age and sex. During the RTT follow-up, kidney transplantation was more frequently performed in non-diabetic patients (25.2%) than in those with diabetes (9.5%). At the end of the study, 8647 ESRD patients with diabetes (55.1% of the entire diabetic population) were dead and cancer was the cause of death for 6.7% of them. Among the 10 518 non-diabetic patients who died (43.6% of the entire non-diabetic population), 13.4% of deaths were related to cancer.

Comparison of the baseline characteristics in patients with type 1 and type 2 diabetes showed that patients with type 1 diabetes were younger and had fewer comorbidities than patients with type 2 diabetes. Moreover, more patients with type 2 diabetes were dead at the end of the study compared to patients with type 1 diabetes. Indeed, the median survival times of patients with type 1 and 2 diabetes were respectively 65 and 43 months (p<0.0001). Cancer was recorded as the cause of death for 6.9% of patients with type 2 diabetes and for 3.9% of patients with type 1 and cardiovascular diseases for 29.2% and 31.7% of patients, respectively.

### Risk of death by cancer

The Fine and Gray survival model, which allows taking into account the competing risk of death by other causes than cancer, was used to evaluate the relationship between the patients’ characteristics and mortality by cancer. In the unadjusted model, diabetes was significantly associated with decreased risk of mortality by cancer (unadjusted HR = 0.63 (95%CI: [0.57–0.69]; p<0.001) (Tables 2 and 3). Similarly, female gender, peritoneal dialysis as first treatment and kidney transplantation during the medical follow-up were all significantly associated with decreased risk of death by cancer (p<0.001). Conversely, age over 60 years, being or having been a smoker, presence of a tumor at RRT initiation, hemoglobin <10g/dl, serum albumin <30g/dl (p<0.001) and respiratory diseases (p = 0.003) were all significantly associated with increased risk of mortality by cancer after taking into account competing events. Moreover, most French regions were associated with increased risk of mortality by cancer and only the region of Pays de la Loire was associated with decreased risk of mortality by cancer (p = 0.010).

**Table 2:** Factors associated with death by cancer in the Fine and Gray analysis.

Patients’ characteristics	Univariate F&G		Multivariate F&G	
	HR (95% CI) *	*p*	HR (95% CI) *	*p*
** Female Gender** *(vs Male)*	0.69 (0.62–0.75)	<0.001	0.81 (0.73–0.90)	<0.001
**Patients’ characteristics at RRT initiation** ^¶^ **:**				
** Age** *(vs <60 years)*				
*[60;75 [*	2.45 (2.14–2.8)	<0.001	1.43 (1.23–1.66)	<0.001
*≥75*	2.30 (2.01–2.63)	<0.001	1.25 (1.08–1.50)	0.004
** Smoking status** *(vs never smoker)*				
*Current smoker*	1.41 (1.21–1.64)	<0.001	1.61 (1.36–1.91)	<0.001
*Former smoker*	1.56 (1.40–1.74)	<0.001	1.44 (1.27–1.63)	<0.001
*Missing*	1.28 (1.14–1.45)	<0.001	1.27 (1.08–1.49)	0.004
** First dialysis modality** ^#^ *(PD vs HD)*	0.60 (0.51–0.71)	<0.001	0.81 (0.68–0.95)	0.010
** Diabetes** *(Yes vs No)*	0.63 (0.57–0.69)	<0.001	0.74 (0.66–0.82)	<0.001
** Active Malignancy** ^‡^ *(vs No)*				
*Yes*	8.75 (8.01–9.57)	<0.001	6.82 (6.19–7.52)	<0.001
*Missing*	0.94 (0.63–1.40)	NS	0.96 (0.51–1.81)	NS
** Respiratory Disease** *(vs No)*				
*Yes*	1.22 (1.07–1.39)	0.003	0.96 (0.84–1.10)	NS
*Missing*	0.81 (0.60–1.12)	NS	1.08 (0.68–1.72)	NS
** Cardio-vascular Disease** ^†^ *(vs No)*				
*Yes*	0.84 (0.77–0.92)	<0.001	0.65 (0.59–0.72)	<0.001
*Missing*	0.46 (0.26–0.79)	0.005	0.46 (0.23–0.93)	0.03
** Cirrhosis** *(vs No)*				
*Yes*	1.12 (0.84–1.49)	NS	1.02 (0.76–1.37)	NS
*Missing*	0.76 (0.54–1.08)	NS	1.20 (0.71–2.03)	NS
** BMI** ^s^ **kg/m²** (vs *[23;25])*				
*<23*	1.04 (0.90–1.20)	NS	1.03 (0.89–1.19)	NS
*>25*	0.77 (0.67–0.89)	<0.001	0.85 (0.74–0.98)	0.028
*Missing*	0.84 (0.73–0.97)	NS	0.97 (0.83–1.13)	NS
** Hemoglobine g/dl** *(vs [10;12])*				
*<10*	1.41 (1.26–1.57)	<0.001	1.13 (1.01–1.27)	0.003
*>12*	0.92 (0.78–1.09)	NS	0.91 (0.76–1.08)	NS
*Missing*	1.17 (1.04–1.32)	0.01	1.24 (1.07–1.44)	0.005
** Albumine g/dl** *(vs ≥30)*				
*<30*	1.55 (1.37–1.76)	<0.001	1.15 (1.0–1.32)	0.036
*Missing*	1.06 (1.04–1.32)	NS	1.04 (0.92–1.17)	NS
** Kidney transplantation during follow-up** *(Yes vs No)*	0.08 (0.06–0.11)	<0.001	0.10 (0.08–0.14)	<0.001

*HR: Hazard Ratio; CI: Confidence Interval

^¶^RRT: Renal Replacement Therapy

^#^PD: Peritoneal Dialysis; HD: Hemodialysis

^‡^Solid tumors and hematological malignancies

^†^Cardio-vascular diseases included: myocardial infarction, arrhythmias, coronary insufficiency, heart failure, lower limbs arteritis, cerebrovascular accident

^s^BMI: Body Mass Index

**Table 3:** Factors associated with death by cancer in the Fine and Gray analysis.

	Univariate F&G		Multivariate F&G	
	HR (95% CI) *	*p*	HR (95% CI) *	*p*
**French regions of residence** *(vs Alsace)*				
*Aquitaine*	1.6 (0,81–3,15)	NS	1.54 (0.78–3.04)	NS
*Auvergne*	3.63 (2,05–6,42)	<0,0001	3.7 (2.05–6.7)	0.000
*Basse-Normandie*	2.32 (1,25–4,31)	0.008	2.16 (1.15–4.06)	0.016
*Bourgogne*	1.99 (1,08–3,66)	0.026	1.88 (1.01–3.5)	0.045
*Bretagne*	2.56 (1,45–4,52)	0.001	2.47 (1.38–4.42)	0.002
*Centre*	1.74 (0,96–3,14)	NS	1.6 (0.87–2.93)	NS
*Champagne-Ardenne*	2.24 (1,24–4,04)	0.007	2.4 (1.31–4.40)	0.004
*Corse*	1.07 (0,35–3,26)	NS	1.23 (0.4–3.82)	NS
*Haute-Normandie*	2.40 (1,31–4,41)	0.005	2.55 (1.37–4.74)	0.003
*Ile de France*	1.32 (0,75–2,32)	NS	1.36 (0.76–2.43)	NS
*Réunion*	.49 (0,16–1,51)	NS	0.59 (0.19–1.83)	NS
*Languedoc-Roussillon*	1.92 (1,09–3,39)	0.025	1.79 (1–3.20)	0.049
*Limousin*	2.07 (1,11–3,83)	0.022	2.24 (1.19–4.23)	0.012
*Lorraine*	2.80 (1,59–4,91)	<0,0001	3.49 (1.96–6.2)	0.000
*Midi-Pyrénées*	2.39 (1,34–4,27)	0.003	2.25 (1.25–4.08)	0.007
*Nord Pas de Calais*	2.30 (1,31–4,02)	0.004	2.55 (1.44–4.51)	0.001
*Pays de Loire*	7.8e-2 (8,9e-31-0,00007)	0.010	9.8e-13 (6.8e-26-14.2)	NS
*Picardie*	2.02 (1,07–3,82)	0.031	1.66 (0.87–3.20)	NS
*Poitou-Charentes*	2.34 (1,21–4,51)	0.012	1.96 (1–3.84)	NS
*PACA*	1.80 (1,02–3,16)	0.042	1.62 (0.91–2.88)	NS
*Rhône-Alpes*	2.21 (1,27–3,85)	0.005	1.5 (0.85–2.64)	NS

*HR: Hazard Ratio; CI: Confidence Interval

Diabetes remained not associated with a significant increased risk of death by cancer even after controlling for the effect of age, gender, smoking, dialysis treatments, clinical characteristics and French regions of residence (adjusted HR = 0.72 95%CI: [0.68–0.95]; p<0.001) (Tables 2 and 3). Similarly, female gender (p<0.001), peritoneal dialysis (p = 0.01), cardiovascular diseases (p<0.001) and kidney transplantation during the RTT follow-up (p<0.001) were associated with decreased risk of mortality by cancer after taking into account the competing risks of death by other causes.

Conversely, age over 60 years, being or having been a smoker, presence of a tumor at RRT initiation (p<0.001), hemoglobin <10g/dl (p = 0.008), serum albumin <30g/dl (p = 0.017) and some French regions (Auvergne, Basse-Normandie, Bourgogne, Bretagne, Haute Normandie, Languedoc-Roussillon, Limousin, Lorraine, Midi-Pyrenees and Nord Pas de Calais) were all significantly associated with increased risk of death by cancer after taking into account the competing risks (Tables 2 and 3). Respiratory diseases, BMI and cirrhosis were not associated with the risk of death by cancer.

After the exclusion in multivariate analysis of: i) transplanted patients; ii) patients with initial active malignancies or iii) patients dead from cancer after 1–2 years of RRT initiation (respectively in SI-SIII Tables in S1 File); there were no notable differences between these results of sensitivity analysis and those of Tables 2 and 3. Moreover, the association between diabetes and decrease risk of death by cancer remained significant even after adjusting for French regions.

### Risk of death by cancer in patients with type 1 or type 2 diabetes

As observed for the entire diabetic population, type 1 (adjusted HR = 0.42 95%CI: [0.27–0.65]) and type 2 diabetes (adjusted HR = 0.86 95%CI: [0.81–0.91]) were both not associated with a significant increased risk of mortality by cancer. In both groups (type 1 and type 2 diabetes), female gender, peritoneal dialysis as first treatment and kidney transplantation during the medical follow-up were all significantly associated with reduced risk of death by cancer (p<0.001). Based on these results, patients with type 1 and type 2 diabetes were considered as a single group for further analyses.

### Effect of diabetes on the risk of death by cancer

**Fig 1:**
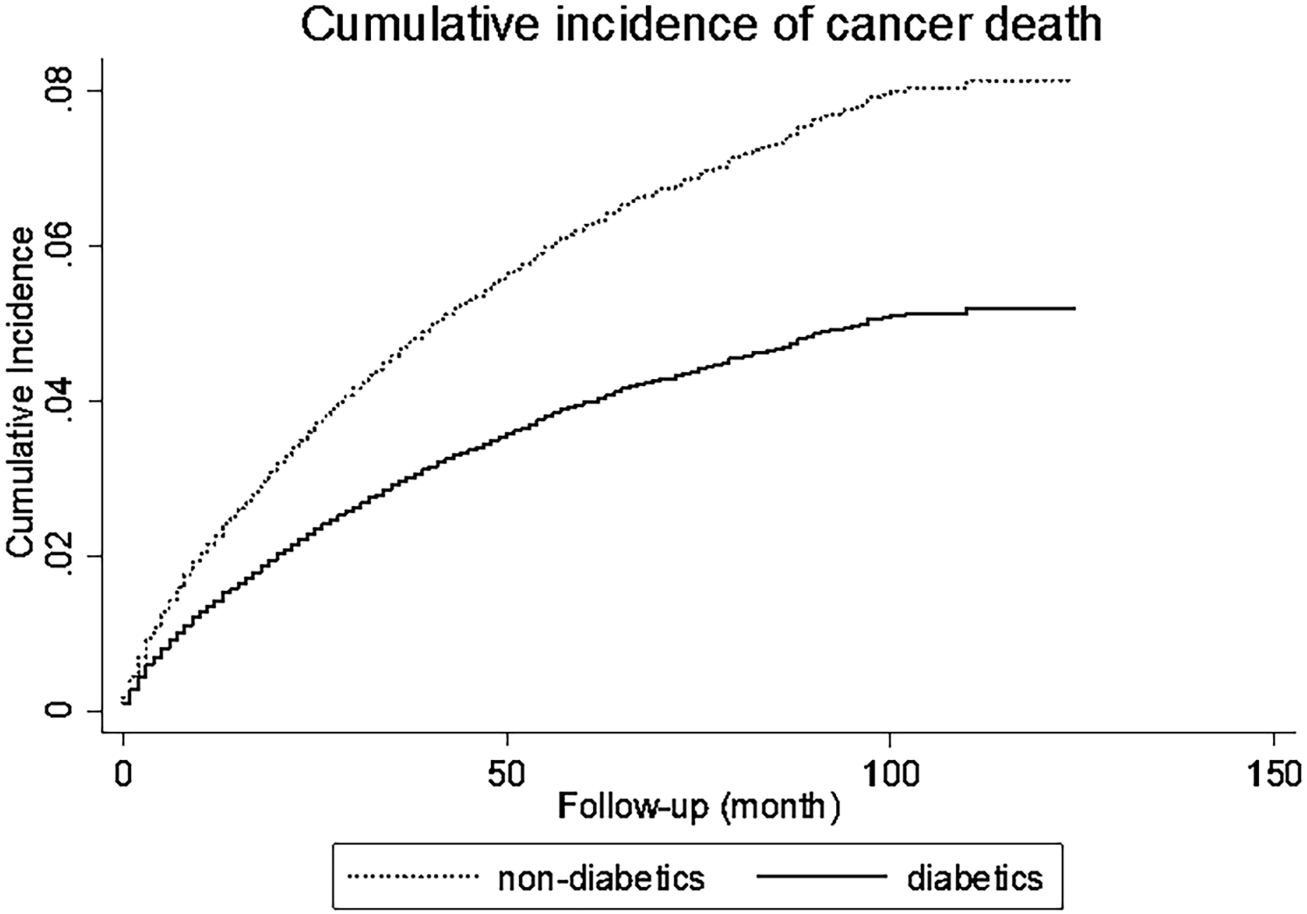
Cumulative Incidence of death by cancer in ESRD patients stratified according to the diabetes status. Diabetic patients (full line) and non-diabetic patients (dot line). The cumulative incidence function (CIF) of death by cancer was estimated after taking into account the competing risk of death by other causes using the Fine and Gray model.

Finally, the cumulative incidence function (CIF) of death by cancer for ESRD patients with or without diabetes was estimated after taking into account the competing risk of death by other causes using the Fine and Gray model (Fig 1). The probability of death by cancer at 100 months after RRT initiation was approximately of 5% in patients with diabetes (solid line) and of 8% in the non-diabetic population (dashed line). However, the survival time of patients with ESRD who died of cancer was not significantly different in the diabetic and non-diabetic populations.

The incidence rate of death by cancer was globally 4.7 per 1 000 patient year. For diabetic patients, the incidence rate was 3.3 per 1 000 patient year and for non-diabetics 5.9 per 1 000 patient year.

## Discussion

Our study using data from a national French registry shows that, differently from the general population, diabetes in patients with ESRD undergoing RRT is not associated with a significant increased risk of death by cancer, after taking into account the competing risk of death by other causes. Moreover, female gender, peritoneal dialysis as first RRT, cardiovascular diseases and kidney transplantation during the clinical follow-up were associated with a significant decreased risk of death by cancer. Conversely, former and current smoking status, serum albumin<30g/dl, hemoglobin<10g/dl, regions of north-west (Bretagne, Haute Normandie, Basse Normandie, Nord Pas de Calais), north-east (Lorraine, Champagne Ardenne), center (Bourgogne, Limousin, Auvergne) and south (Midi Pyrenees, Languedoc Roussillon) of France were significantly associated with increased risk of cancer death.

To our knowledge, this is the first population-based study with such a large cohort of incident patients with ESRD (n = 39 811). These data were extracted from the REIN registry that includes all patients starting dialysis in most regions of France. No patient was lost during follow-up. Therefore, our data can be considered to be highly reliable and complete.

Our study has some limitations. Ideally, the association between diabetes and cancer should be analyzed in terms of tumor incidence and not only of mortality. However, our data were extracted from the REIN registry in which cancer incidence is not a mandatory field, differently from the cause of death. Moreover, in the REIN, there is a lack of information related to the treatment of cancer administered in patients and grades or types of cancer. In addition, in France there is no national cancer registry that we could use to study tumor incidence. Another limitation might concern the relative heterogeneity in the burden of diabetes-related comorbidities. Indeed in the REIN registry, data relative to the diabetes history before the ESRD stage are missing (duration, severity, complications…).

This study confirms, as previously described in the annual reports of the US Renal Data System [1] or in the French REIN registry [17], that the mortality rate is higher in ESRD patients with diabetes than in those without diabetes. However, diabetic patients had 30% lower risk of death by cancer compared to the non-diabetic population. In addition, at dialysis initiation, the presence of a cancer was less frequently recorded for patients with diabetes than for those without diabetes after adjustment for age and sex. These findings are consistent with previous studies reporting lower cancer rate in diabetic patients with chronic kidney disease [5] or with ESRD [10–13]. For instance, *Maisonneuve et al*., found that although the overall risk of cancer was higher in patients with ESRD than in the general population in Europe, this increase was much lower in patients with diabetic nephropathy than in people with other ESRD etiologies (risk of cancer in ESRD patients with diabetic nephropathy: HR = 0.8; 95%CI: [0.8–0.9] versus risk for the other etiologies such as glomerulonephritis: HR = 1.3; 95%CI: [1.2–1.4]; congenital: HR = 1.4; 95%CI: [1.0–2.1]; toxic nephropathies: HR = 1.7; 95%CI: [1.5–1.8]) [12]. *Stewart et al*. showed that the occurrence of kidney (HR = 0.71; 95%CI: [0.60–0.85]) and bladder cancer (HR = 0.60; 95%CI: (0.60–0.85)) was significantly reduced among patients with diabetic nephropathy compared with other nephropathies [11]. *Denton et al*., found that diabetes was inversely associated with the presence of renal adenomas in patients with ESRD before kidney transplantation (HR = 0.1; 95%CI: [0.1–0.8]) [10]. Finally, *Wong et al*. observed that mild to moderate chronic kidney disease did not increase the risk of cancer in patients with type 2 diabetes [5]. However, these studies have several limitations. Some used data issued from different registries and of variable quality [11–13]. Indeed, when data were not available in the national registries, information was extracted from regional registries or from neighboring countries. In other studies, only one type of cancer was specifically studied [10, 11]. Moreover, unlike our study, they did not take into account the patients’ clinical characteristics (comorbidities, BMI, serum albumin and hemoglobin) [5, 12, 13]. This is important because patients with ESRD and diabetes often have several comorbidities that could lead to premature death and also to higher mortality rates compared with ESRD patients without diabetes. For this reason, the competing risks of death should be taken into account when analyzing the risk of death by cancer in patients with ESRD, especially if also diabetics.

Surprisingly, kidney transplantation during the follow-up was not associated with increased risk of death by cancer, possibly due to the rigorous follow-up of this patients’ category, leading to earlier diagnosis and, perhaps, lower cancer mortality. In our study, starting first renal replacement therapy with a HD was associated with increased risk of death by cancer. Some authors showed that patients on PD had better immunologic statements which preserve them against environmental attacks and cancer development [15]. Moreover, we observed that low levels of hemoglobin and albumin were also associated with an increased risk of death by cancer. These results joined previous studies which reported that anemic patients suffering of cancer had significant reduction in survival [31] and that albuminuria was both associated with an increased risk of cancer death from all causes [32, 33] and related to cancer incidence [34]. Hillege et al., found that death caused by malignancies explained the increase of non-cardiovascular mortality risk [33] which could explain why the presence of cardio-vascular disease at dialysis initiation was not associated with an increased risk of death by cancer. The relation between French regions and the mortality by cancer is coherent with results given by the INCa: Institut National du Cancer, which showed two clusters of regions from north-west to north-east and from north to center where the incidence of cancer and the mortality by cancer is higher than the other regions [35].

In summary our data indicate that the frequency of cancer at dialysis initiation and the risk of death by cancer were reduced in patients with ESRD and diabetes compared to non-diabetic patients. However, in the general population, cancer incidence is higher in patients with diabetes compared to people without diabetes [18–20]. Different hypotheses could explain these discordant observations. First, diabetic patients with chronic kidney disease could develop cancer and die before the terminal stage of the kidney disease; or the association of cancer and diabetes could be considered as a barrier to dialysis initiation. This may explain the lower rate of cancer in patients with diabetes than in those without diabetes at the moment of their inclusion in the REIN registry. Second, diabetic individuals in dialysis need less insulin than those with normal kidney function [24, 25]. Indeed, renal function impairment is associated with reduced insulino-resistance [24, 25] and improved insulin clearance [25]. As insulin is one of the growth factors involved in cancer development [19–21], these effects could contribute to the reduced cancer rate in dialyzed ESRD patients with diabetes. Third, diabetic patients with kidney disease usually receive long-term treatments with renin angiotensin system blockers to slow renal failure progression before dialysis and for cardiologic indications after starting dialysis [26–28]. Blocking the renin-angiotensin system is especially effective in protecting against diabetic nephropathy progression [27, 28]. In addition, recent studies suggest that renin angiotensin system blockers can also decrease cancer growth [29, 30]. Indeed, a study on a large Taiwanese cohort reported that cancer incidence was reduced in patients with hypertension and several comorbidities (such as diabetes and chronic renal disease) treated with renin angiotensin system blockers compared with similar patients who did not receive these treatments [26]. Therefore, it could be hypothesized that, besides their renoprotective effects, renin-angiotensin system blockers might act also as a protective agent against cancer development in patients with ESRD and diabetes [29]. However, studies on cancer incidence and not only cancer mortality need to be carried out to support/rule out these hypotheses.

In conclusion, by analyzing the risk of death by cancer after taking into account competing events, our study shows that in patients with ESRD, diabetes as well as female gender, peritoneal dialysis, cardiovascular diseases and kidney transplantation during the medical follow-up are associated with reduced risk of cancer death. However, in patients with ESRD and diabetes, who generally have several comorbidities, the risks of death by other causes than cancer is higher than in patients without diabetes. Studies examining the incidence of cancer in patients with ESRD are needed to evaluate the potential association between diabetes and specific malignancies in this population.

## Supporting Information

S1 File **SI Table.** Factors associated with death by cancer in the Fine and Gray analysis, after the exclusions of transplanted patients (n = 32 253). **SII Table.** Factors associated with death by cancer in the Fine and Gray analysis, after the exclusion of patients with initial active malignancies (n = 35 864). **SIII Table.** Factors associated with death by cancer in the Fine and Gray analysis; after the exclusion of patients dead from cancer after 1–2 years of RRT initiation (n = 38 619).
(DOCX)
