# Supplementary material for: Type 1 and Type 2 Diabetes and Cancer Mortality in the 2002-2009 Cohort of 39 811 French Dialyzed Patients
Source: PLoS One. 2015 May 12;10(5):e0125089. doi: 10.1371/journal.pone.0125089 (PMC4428826; doi:10.1371/journal.pone.0125089)
Supplement: S1 File — SI Table. Factors associated with death by cancer in the Fine and Gray analysis, after the exclusions of transplanted patients (n = 32 253). SII Table. Factors associated with death by cancer in the Fine and Gray analysis, after the exclusion of patients with initial active malignancies (n = 35 864). SIII Table. Factors associated with death by cancer in the Fine and Gray analysis; after the exclusion of patients dead from cancer after 1–2 years of RRT initiation (n = 38 619). (DOCX) [file pone.0125089.s001.docx]

**Supporting Information**

**Table S1. Factors associated with death by cancer in the Fine and Gray analysis; after the exclusions of transplanted patients (n=32 253)**

|  | **Multivariate F&G (n=32 253)** | | |
| --- | --- | --- | --- |
|  | **HR*** | **CI (95%)** | **p** |
| **Female Gender** *(vs Male)* | 0.82 | 0.74-0.91 | <0.001 |
| **Patients’ characteristics at RRT initiation**^¶^**:** |  |  |  |
| **Age** *(vs <60 years)* |  |  |  |
| *[60;75[* | 1.34 | 1.16-1.55 | <0.0001 |
| *≥75* | 1.19 | 1.02-1.38 | 0.026 |
| **Smoking status** *(vs never smoker)* |  |  |  |
| *Current smoker* | 1.61 | 1.35-1.91 | <0.0001 |
| *Former smoker* | 1.46 | 1.28-1.65 | <0.0001 |
| *Missing* | 1.29 | 1.08-1.51 | 0.003 |
| **First dialysis modality**^#^ *(PD vs HD)* | 0.81 | 0.68-0.96 | 0.013 |
| **Diabetes** *(Yes vs No)* | 0.74 | 0.66-0.82 | <0.0001 |
| **Active Malignancy**^‡^ *(vs No)* |  |  |  |
| *Yes* | 6.73 | 6.10-7.42 | <0.0001 |
| *Missing* | 0.97 | 0.51-1.85 | NS |
| **Respiratory Disease** *(vs No)* |  |  |  |
| *Yes* | 0.97 | 0.84-1.11 | NS |
| *Missing* | 1.04 | 0.65-1.67 | NS |
| **Cardio-vascular Disease**^†^ *(vs No)* |  |  |  |
| *Yes* | 0.64 | 0.58-0.71 | <0.0001 |
| *Missing* | 0.42 | 0.20-0.88 | 0.021 |
| **Cirrhosis** *(vs No)* |  |  |  |
| *Yes* | 0.97 | 0.72-1.31 | NS |
| *Missing* | 1.23 | 0.72-2.10 | NS |
| **BMI**^s^ **kg/m²** (vs *[23;25])* |  |  |  |
| *<23* | 1.03 | 0.89-1.19 | NS |
| *>25* | 0.85 | 0.73-0.98 | 0.023 |
| *Missing* | 0.96 | 0.82-1.12 | NS |
| **Hemoglobin g/dl** *(vs [10;12])* |  |  |  |
| *<10* | 1.13 | 1.01-1.27 | 0.036 |
| *>12* | 0.92 | 0.77-1.09 | NS |
| *Missing* | 1.24 | 1.07-1.44 | 0.005 |
| **Albumin g/dl** *(vs ≥30)* |  |  |  |
| *<30* | 1.15 | 1.0-1.31 | 0.045 |
| *Missing* | 1.05 | 0.91-1.17 | NS |

*^*^ Analysis adjusted on French regions of residence. ^*^HR: Hazard Ratio; CI: Confidence Interval*

^¶^*RRT: Renal Replacement Therapy*

^#^*PD: Peritoneal Dialysis; HD: Hemodialysis*

^‡^*Solid tumors and hematological malignancies*

^†^*Cardio-vascular diseases included: myocardial infarction, arrhythmias, coronary insufficiency, heart failure, lower limbs arteritis, cerebrovascular accident*

^s^*BMI: Body Mass Index*

**Table S2. Factors associated with death by cancer in the Fine and Gray analysis; after the exclusion of patients with initial active malignancies (n=35 864):**

|  | **Multivariate F&G (n=35 864)** | | |
| --- | --- | --- | --- |
|  | **HR*** | **CI (95%)** | **p** |
| **Female Gender** *(vs Male)* | 0.64 | 0.56-0.74 | <0.0001 |
| **Patients’ characteristics at RRT initiation**^¶^**:** |  |  |  |
| **Age** *(vs <60 years)* |  |  |  |
| *[60;75[* | 1.87 | 1.53-2.29 | <0.0001 |
| *≥75* | 1.78 | 1.44-2.20 | <0.0001 |
| **Smoking status** *(vs never smoker)* |  |  |  |
| *Current smoker* | 2.14 | 1.74-2.63 | <0.0001 |
| *Former smoker* | 1.68 | 1.42-1.98 | <0.0001 |
| *Missing* | 1.22 | 0.98-1.53 | NS |
| **First dialysis modality**^#^ *(PD vs HD)* | 0.78 | 0.64-0.96 | 0.017 |
| **Diabetes** *(Yes vs No)* | 0.71 | 0.62-0.81 | <0.0001 |
| **Respiratory Disease** *(vs No)* |  |  |  |
| *Yes* | 0.96 | 0.81-1.15 | NS |
| *Missing* | 1.67 | 1.01-2.78 | NS |
| **Cardio-vascular Disease**^†^ *(vs No)* |  |  |  |
| *Yes* | 0.62 | 0.54-0.70 | <0.0001 |
| *Missing* | 0.37 | 0.16-0.87 | 0.023 |
| **Cirrhosis** *(vs No)* |  |  |  |
| *Yes* | 1.05 | 0.74-1.49 | NS |
| *Missing* | 0.92 | 0.50-1.67 | NS |
| **BMI**^s^ **kg/m²** (vs *[23;25])* |  |  |  |
| *<23* | 1.05 | 0.86-1.27 | NS |
| *>25* | 0.91 | 0.76-1.10 | NS |
| *Missing* | 0.90 | 0.73-1.12 | NS |
| **Hemoglobin g/dl** *(vs [10;12])* |  |  |  |
| *<10* | 1.11 | 0.95-1.29 | NS |
| *>12* | 1.00 | 0.81-1.24 | NS |
| *Missing* | 1.20 | 0.99-1.47 | NS |
| **Albumin g/dl** *(vs ≥30)* |  |  |  |
| *<30* | 0.99 | 0.82-1.20 | NS |
| *Missing* | 0.94 | 0.80-1.11 | NS |
| **Kidney transplantation during follow-up** *(Yes vs No)* | 0.09 | 0.06-0.13 | <0.0001 |

*^*^ Analysis adjusted on French regions of residence.* *HR: Hazard Ratio; CI: Confidence Interval*

^¶^*RRT: Renal Replacement Therapy*

^#^*PD: Peritoneal Dialysis; HD: Hemodialysis*

^†^*Cardio-vascular diseases included: myocardial infarction, arrhythmias, coronary insufficiency, heart failure, lower limbs arteritis, cerebrovascular accident*

^s^*BMI: Body Mass Index*

**Table S3. Factors associated with death by cancer in the Fine and Gray analysis; after the exclusion of patients dead from cancer after 1-2 years of RRT initiation (n=38 619)**

|  | **Multivariate F&G (n=38 619)** | | |
| --- | --- | --- | --- |
|  | **HR*** | **CI (95%)** | **p** |
| **Female Gender** *(vs Male)* | 0.72 | 0.61-0.85 | <0.0001 |
| **Patients’ characteristics at RRT initiation**^¶^**:** |  |  |  |
| **Age** *(vs <60 years)* |  |  |  |
| *[60;75[* | 1.6 | 1.25-1.97 | <0.0001 |
| *≥75* | 1.22 | 0.96-1.56 | NS |
| **Smoking status** *(vs never smoker)* |  |  |  |
| *Current smoker* | 2.11 | 1.67-2.67 | <0.0001 |
| *Former smoker* | 1.51 | 1.25-1.82 | <0.0001 |
| *Missing* | 1.10 | 0.83-1.45 | NS |
| **First dialysis modality**^#^ *(PD vs HD)* | 0.70 | 0.54-0.90 | 0.007 |
| **Diabetes** *(Yes vs No)* | 0.70 | 0.59-0.83 | <0.0001 |
| **Active Malignancy** ^‡^ *(vs No)* |  |  |  |
| *Yes* | 2.97 | 2.49-3.53 | <0.0001 |
| *Missing* | 0.70 | 0.24-1.98 | NS |
| **Respiratory Disease** *(vs No)* |  |  |  |
| *Yes* | 1.02 | 0.83-1.26 | NS |
| *Missing* | 1.94 | 1.03-3.63 | 0.039 |
| **Cardio-vascular Disease**^†^ *(vs No)* |  |  |  |
| *Yes* | 0.62 | 0.53-0.73 | <0.0001 |
| *Missing* | 0.58 | 0.21-1.60 | NS |
| **Cirrhosis** *(vs No)* |  |  |  |
| *Yes* | 1.26 | 0.85-1.88 | NS |
| *Missing* | 1.12 | 0.50-2.51 | NS |
| **BMI**^s^ **kg/m²** (vs *[23;25])* |  |  |  |
| *<23* | 0.91 | 0.73-1.14 | NS |
| *>25* | 0.97 | 0.79-1.20 | NS |
| *Missing* | 0.72 | 0.57-0.93 | 0.011 |
| **Hemoglobin g/dl** *(vs [10;12])* |  |  |  |
| *<10* | 0.96 | 0.80-1.15 | NS |
| *>12* | 1.07 | 0.84-1.37 | NS |
| *Missing* | 1.23 | 0.97-1.56 | NS |
| **Albumin g/dl** *(vs ≥30)* |  |  |  |
| *<30* | 0.92 | 0.73-1.15 | NS |
| *Missing* | 0.95 | 0.79-1.15 | NS |
| **Kidney transplantation during follow-up** *(Yes vs No)* | 0.12 | 0.07-0.18 | <0.0001 |

*^*^Analysis adjusted on French regions of residence. HR: Hazard Ratio; CI: Confidence Interval*

^¶^*RRT: Renal Replacement Therapy*

^#^*PD: Peritoneal Dialysis; HD: Hemodialysis*

^‡^*Solid tumors and hematological malignancies*

^†^*Cardio-vascular diseases included: myocardial infarction, arrhythmias, coronary insufficiency, heart failure, lower limbs arteritis, cerebrovascular accident*

^s^*BMI: Body Mass Index*
